# Predictors of smartphone addiction and its effect on quality of life: a cross-sectional study among the young adults in Bangladesh

**DOI:** 10.3389/fdgth.2025.1351955

**Published:** 2025-02-12

**Authors:** Zubair Ahmed Ratan, Anne-Maree Parrish, Mohammad Saud Alotaibi, Hassan Hosseinzadeh

**Affiliations:** ^1^ Faculty of the Arts, Social Sciences and Humanities, School of Health and Society, University of Wollongong, Wollongong, NSW, Australia; ^2^ Department of Social Work, College of Social Sciences, Umm Al-Qura University, Mecca, Saudi Arabia

**Keywords:** smartphone addiction, predictors, quality of life, Bangladesh, compulsive usage

## Abstract

The enigma of smartphone addiction (SA) has plagued academics for the last decade, now scholars believed this behaviour might affect physical and mental wellbeing. SA has become a complex problem, yet to date, there is limited research investigating the predictors of SA and its effect on “health-related quality of life (HRQoL)”. This study aimed to address this gap. The data was gathered from a convenience sample of 440 young adults completed between July 2021 and February 2022 through online survey in Bangladesh. On Logistic regression, after controlling for socio-demographic variables; friend support, process, social and compulsive usage were determined as significant predictors of SA. Those who were smartphone addicted were more presumably to have a lower quality of life. This study has significant implications for designing prevention pro-grams and policy development in relation to predictors of SA and its effect on HRQoL.

## 1 Introduction

In 2007, Steve Jobs, co-founder of Apple, unveiled a revolutionary product that brought remarkable changes in the phone industry bringing the “smartphone” into existence (1). Since then, it become an indispensable component of modern lifestyle. It has changed the entire field of communication. The Smartphone affords easy internet access, is capable of impressive levels of data analysis and contains microphones, a camera, and lights (2, 3). In 2018, there were around three billion users of smartphone globally (4) and probably, it will reach over seven billion by 2026 (5). People use smartphones for social networking, gaming, shopping, taking photos, listening to music or watching movies (6). Despite its benefits, overuse of these devices can lead to SA (7) and SA is a growing public health concern all over the world (8). Different terminology was used to describe SA such as “problematic mobile phone use” “mobile phone addiction”, “smartphone overuse”, “(9), and “excessive use of smartphones” (10) and “addiction proneness” (11), and all representing a behavioral addiction. Till now, two types of behavioural addictions, gambling, and internet gaming disorders have been added to the “Diagnostic and Statistical Manual of Mental Disorders”, Fifth Edition (DSM-5) (12). Ching, Yee (13), defined SA as “mainly characterized by excessive or poorly controlled preoccupations, usage or behaviour regarding smartphone use; to the extent that individuals neglect other areas of life”. In many instances, smartphone users look at their devices while driving or walking (14) creating additional opportunities for harm. In addition SA use can make individuals “nomophobic”, which was narrated by Bhattacharya, Bashar (15) as “a psychological condition when people have a fear of being detached from mobile phone connectivity” [or fear of missing out (FOMO)].

Several factors are discussed to be responsible for SA. One recent study found that habitual use could be transformed into SA (16). Another found, increased habits of checking phone's notifications can lead to increased problematic smartphone use (17). Smartphone has become a popular tool for process use (e.g., entertainment) and social use (e.g., communication) (18). Process use has been identified as gratifying effects of consuming media (19), whereas social use can give pleasurable experience to the participants through social interaction (18). These user patterns may trigger the “reward system of addictive behaviors” (20). A recent survey revealed that 58.7% of the world's population use social media (21). Previous studies reported process and social usage as a strong determinant of SA (18). Furthermore, compulsive behaviour has been identified as a diagnostic criteria of SA (22) and it is positively associated with SA. O'Guinn and Faber (23) defined compulsive usage as “an uncontrollable drive or desire to obtain, use, or experience a feeling, substance, or activity that leads the individual to repeatedly engage in behavior that will ultimately cause harm to the individual and/or others”. Previous study also revealed that the compulsive behaviors are more likely to result in negative psychological distress outcomes, including stress and depression, which is also relevant to smartphone addition (24). Previous studies found that lower levels of physical activity, musculoskeletal pain, or poor sleep were associated to SA (25–27). Studies also revealed that SA was directly correlated to anxiety, stress and negative emotions (28, 29). Moreover, SA can reduce face-to-face communication (30, 31) and lead to social isolation (32, 33). Previous studies emphasized that SA was related to poor HRQoL (34, 35), and HRQoL includes the “physical, mental, social, emotional, and behavioral” components of well-being (36).

In today's world, the smartphone has become an essential tool from highly developed to the less developed countries (37). Bangladesh represents one of the developing countries that has been digitalized by increasing internet use, at present, more than 176 million mobile phone users in Bangladesh (38). To our best knowledge, no previous study has explored the predictors of SA and its impact on HRQoL among young adults in Bangladesh. To reduce this gap, the current study aimed:

(1) To what extent different form of smartphone usage and social support can predict and determine SA among young adults in Bangladesh?
(2) To identify the effect of SA on HRQoL among young adults in Bangladesh.

## 2 Materials and methods

### 2.1 Study design and data collection

This study was cross-sectional in design and conducted among the young adults in Bangladesh. In order to qualify for participation, applicants must be aged between 18 and 32 years old, fluent in spoken and written Bengali, and used a smartphone for at least one year. Participants who were under the age of 18 or over the age of 32, unable to read or write in Bengali, or who had a mobile phone that was less than one year old were excluded from the study.

The data was collected via an online survey which was administered through Qualtrics from July 2021 to February 2022. This study was advertised through flyers, social media platforms and emails. Participants were recruited through the advertisements, as the online survey link was attached to the study advertisement. “Participant Information Sheet (PIS)”and “Participant Consent Form (PCF)” were included with the online survey, where participants could read PIS and accept PCF before seeing and completing the survey. This current study received approval from the “University of Wollongong Human Research Ethics Committee (HREC)” in July 2021 (Reference number- 2021/059).

### 2.2 Sample size

A power analytic methodology was utilized to calculate an appropriate sample size for the quantitative phase of the study. According to Cohen's formula, 304 respondents would give a 95% chance of identifying a correlation of less than 0.10 at the 0.05 level with a maximum of ten predicting variables in the model, allowing for a 15% possibility of incomplete surveys (39). The least effect that would be significant to detect for the purpose of this study was an effect size of 0.10. In total, 440 eligible young adults participated in the study.

### 2.3 Study measures

Sociodemographic data included sex; age; gainfully employment status; family income; marital status; family size; semesters of study.

#### 2.3.1 Smartphone addiction

SA was assessed through the “Smartphone Addiction Scale (SAS-SV)” which is an internationally recognized scale (40). It is a 10-item questionnaire, scoring from 1 = “Strongly disagree” to 6 = “Strongly agree” on a Likert scale. The total score for SAS-SV ranges from 10 to 60, a higher score indicates more problematic smartphone use. For males, the cut-off value of SA is 31 but it is 33 for females.

#### 2.3.2 The social and process usage of smartphone

The social and process usage of smartphone had been assessed by the social and process usage scales which was adapted from Chua, Goh (41). It consists of 12 items (five items for social usage and seven items for process usage) that include a Likert scale ranging from “strongly disagree = 1” to “strongly agree = 5”.

#### 2.3.3 Compulsive usage of smartphone

To determine the compulsive usage of smartphones, the research used the “compulsive usage scale”. it was developed by Lee, Chang (42) and has been modified by Dinesh and Arulchelvan (43). It consists of seven items (Cronbach's alpha = 0.84) with response options ranging from “strongly disagree = 1” to “strongly agree = 5”.

#### 2.3.4 The quality of life (WHOQOL-BREF)

The 26 items “World Health Organization Quality of Life Questionnaire (WHOQOL-BREF)” was used to measure “physical health (7 questions)”, “psychological health (6 questions)”, “environmental health” “(8 questions)” and “social relationships (3 questions)”. A “5-point scale” was used to evaluate each item and domain scores were transformed into a percentage “0–100; midpoint 50”.

### 2.4 Translation data collection tools

There are many different approaches to survey translation, such as forward-only translation, forward-only translation with testing, back-translation, back-translation with a monolingual test, back-translation with a bilingual test, and back-translation with both a monolingual test and a bilingual test (44). The technique of back-translation, which was proposed by Brislin (45), was utilized to translate the study instruments into Bengali language, a predominant language spoken in Bangladesh. The instruments which were only available in the English language were translated into the Bengali language and then back into the English language. In order to check and ensure the consistency of the translations, it was carried out by two academics who were fluent in both Bengali and English. First the measures were translated from English into Bengali. Then it was translated back English by another academic. After that, the two academics compared the English versions of the measures to address any differences, and then they finalized the translated versions.

### 2.5 Data analysis

Data were subjected to analyse using “IBM SPSS Statistics Version 27.0.”. Univariate statistics were performed to determine the prevalence of SA and describe sociodemographic. Onley 15 cases with Z-scores higher than 3 or less than −3 in each of the study scales were identified as outliers and removed from the analysis (46).

The means of independent variables (smartphone usage and perceived social supports; quality of life scales) were compared by independent *t*-tests to determine whether there was any significant difference between the smartphone addicted and non-addicted groups.

“Forward stepwise logistic regression” analyses were done to find predictors of SA among the independent variables. The “forward stepwise regression model” adds the most significant variables into the model until none of the remaining variables can improve the model (47).

## 3 Results

### 3.1 Comparison analysis

As shown in Table 1, smartphone addicted participants had higher scores for compulsive usage (M = 40.44, SD = 9.03), process usage (M = 25.16, SD = 3.37), and social usage (M = 19.18, SD = 3.00), but lower scores for family support (M = 19.27, SD = 7.03), friend support (M = 18.04, SD = 6.57), and significant other support (M = 19.27, SD = 6.49) than non-addicted participants.

**Table 1:** Comparison of process usage, social usage, compulsive usage, significant other support, family support, and friends support between smartphone addicted and non-addicted groups.

	*n*	Score range	Non smartphone addicted		Smartphone addicted		t	*P*
			M	(SD)	M	(SD)		
Process usage	428	7–35	23.47	(3.78)	25.16	(3.37)	4.80	0.001
Social usage^a^	434	5–25	18.09	(3.32)	19.18	(3.00)	3.43	0.001
Compulsive usage	440	13–65	34.27	(9.24)	40.44	(9.03)	6.91	0.001
Significant other support	440	4–28	19.77	(6.22)	19.27	(6.49)	−2.46	0.014
Family support^a^	440	4–28	20.85	(5.70)	19.27	(7.03)	−2.58	0.010
Friend support^a^	440	4–28	19.82	(5.49)	18.04	(6.57)	−3.07	0.002

*M*, mean, SD, standard deviation, A *p*-value ≤ 0.05 was considered significant, *n* = number of valid cases (after excluded outliers cases).

^a^ The t and df were adjusted because variances were not equal according to Levene's Test.

### 3.2 Univariate forward stepwise logistic regression

After performing univariate logistic regression analysis (Table 2), we found that social usage, process usage, compulsive usage, significant other support family support and friend support were the significant predictors of smartphone addiction among the participants. Process usage (OR = 1.14, 95% CI: 1.08–1.21), social usage (OR = 1.11 95% CI: 1.04–1.18), and compulsive usage (OR = 1.08, 95% CI: 1.05–1.10) increased the possibility of smartphone addiction by 14%, 11%, and 8% respectively. On the other hand, significant other support (OR = 0.96, 95% CI: 0.93–0.99), friend support (OR = 0.95, 95% CI: 0.92–0.98), and family support (OR = 0.96, 95% CI: 0.93–0.99) decreased the possibility of smartphone addiction by 4%, 4% and 5% respectively.

**Table 2:** Forward stepwise logistic regression analysis of associations of process usage, social usage, compulsive usage, significant other support, family support, and friends support, family support, and friends support with smartphone addiction.

	*n*	*OR*	95% CI		*P*
			Lower	Upper	
Process usage	428	1.14	1.08	1.21	0.001
Social usage	434	1.11	1.04	1.18	0.001
Compulsive usage	440	1.08	1.05	1.10	0.001
Significant other support	440	0.96	0.93	0.99	0.015
Family support	440	0.96	0.93	0.99	0.015
Friend support	440	0.95	0.92	0.98	0.004

*OR*, odds ratio; *CI*, confidence interval, A *p*-value ≤ 0.05 was considered significant, *n* = number of valid cases (after excluded outliers cases).

### 3.3 Forward stepwise multivariate logistic regression

A forward stepwise multivariate logistic regression analysis was performed to assess the significant predictors of smartphone addiction among the participants. The analysis suggested that process usage, social usage and compulsive usage were the predictors of smartphone addiction in the presence of each other (see Table 3).

**Table 3:** Forward stepwise logistic regression of analysis of associations of process usage, social usage, compulsive usage, significant other support, family support, and friends support with smartphone addiction.

	*OR*	95% CI		*P*
		Lower	Upper	
Process usage	1.07	1.00	1.15	0.035
Social usage	1.10	1.03	1.19	0.006
Compulsive usage	1.08	1.05	1.11	0.001
Friend support	0.92	0.89	0.96	0.001

Model chi-square = 81.02, df = 4, *p* ≤ 0.001, R Square = 0.23.

*OR*, odds ratio; *CI*, confidence interval, A *p*-value ≤ 0.05 was considered significant, 15 cases were identified as outliers and excluded from the analysis.

### 3.4 Multivariate forward stepwise logistic regression controlling socio-demographic variables

As stated in Table 4, while controlling for the socio-demographic variables, social usage, process usage, friend support and compulsive usage were still significant predictors of smartphone addiction. Being male, having a younger age ≤ 25), and being unemployed were strong predictors of smartphone addiction. More precisely, male, younger and unemployed participants were 1.92–2.52 times more likely to be addicted.

**Table 4:** Forward stepwise logistic regression analysis of associations of process usage, social usage, compulsive usage, and friends support with smartphone addiction controlling for the potential confounding effects of socio-demographic variables.

	*OR*	95% CI		*p*
		Lower	Upper	
Process usage	1.08	1.00	1.16	0.029
Social usage	1.09	1.01	1.17	0.020
Compulsive usage	1.09	1.06	1.12	0.001
Friend support	0.93	0.89	0.97	0.001
Gender				
Male vs. Female	1.92	1.22	3.02	0.005
Age				
≤25 vs. ≥31	2.52	1.53	4.15	0.001
Occupation status				
Unemployed vs. Employed	2.04	1.00	4.14	0.049

Model chi-square = 104.93, df = 7, *p* ≤ 0.001, R Square = 0.29.

*OR*, odds ratio; *CI*, confidence interval, A *p*-value ≤ 0.05 was considered significant.

### 3.5 Assessing the effect of smartphone addiction on quality of life

As presented in Table 5, the individuals who were addicted to smartphone were more likely to have a reduced quality of life assessed by WHOQOL-BREF in terms of Physical health, psychological health, social relationships and environmental health compared to their none addicted counterparts.

**Table 5:** Comparison of quality of life between smartphone addicted and non-addicted groups.

	*n*	Score range	Non smartphone addicted		Smartphone addicted		t	*p*
			M	(SD)	M	(SD)		
Physical Health	438	9–45	25.80	(3.90)	24.81	(3.92)	−2.56	0.011
Psychological	438	6–30	20.34	(4.34)	19.16	(3.88)	−2.97	0.003
Social Relationships	435	3–15	11.24	(2.09)	10.48	(2.14)	−3.62	0.001
Environment	439	8–40	25.25	(4.73)	23.94	(4.65)	−2.87	0.004

M, mean; SD, standard deviation, A *p*-value ≤ 0.05 was considered significant, *n* = number of valid cases (after excluded outliers cases).

## 4 Discussion

This study revealed that “process usage and social usage” were significant predictors of SA, which is similar to previous studies (18, 48). This might be explained by the fact that process usage and social usage opened a newer horizon in the entertainment and communication industries that leads pleasurable experience. For instance, revealing new features, or checking new notifications in social media can stimulate the “reward center” of the brain (49), leading to increased recurrence of behaviour (50). This pattern of use may result in habitual use, which is ultimately difficult to control. It is worth noting that at present different musical applications, social media platforms have become the prime source of entertainment especially for young people (51, 52), and regular notifications from these sources can provoke the “Fear of missing out (FoMO)” which can lead to SA (53). In addition, compulsive behaviour has been recognized as one of the core components of addiction (54, 55), and interestingly, those who are addicted to their devices are more likely to lose self-control or self-regulation due to compulsive usage (56).

Our results also reported that SA can reduce quality of life in all four domains among smartphone addicted group compared to the non-addicted group. These results in line with the findings of previous studies (57–59) which also reported a negative correlation between SA and the quality of life. Those who are addicted to SA, may have a physical connection to their device and carry their device with them all of the time (60). This phenomenon was explained by Natasha et al. through attachment theory (61), a constant connectivity with their devices may be used to maintain a sense of security and inhibit the feelings of insecurity, ultimately, this pattern of behaviour maintains persistent brain use. There is growing evidence that SA can deteriorate physical and mental health (62–64), in addition, insomnia and poor sleep quality has been identified as a negative consequences of SA (65, 66). Previous studies also suggested that SA is associated with Attention-deficit hyperactivity disorder (ADHD) (67, 68). Moreover, previous research reported that SA is associated with social isolation and loneliness (32, 69). Ultimately, all these conditions diminish the quality of life those who are addicted to their devices.

## 5 Conclusions

The results of this study provide invaluable information to understand the effects of SA, which may have significant implications for developing interventions to reduce smartphone addiction. Uninstalling applications from phones, adding time limits or limited internet access during work or sleep may be helpful in reducing smartphone use among the youth. This current study has several limitations that deserve to be mentioned. It is a cross-sectional study by design, so it is difficult to determine a causal relationship. Moreover, data were self-reported which can create recall bias. In addition, the data was collected using a convenience sample, so these findings may not be generalized for the wider Bangladeshi population of youth.

## Data Availability

The raw data supporting the conclusions of this article will be made available by the authors, without undue reservation.
